# Targeting BCMA in Multiple Myeloma: Advances in Antibody-Drug Conjugate Therapy

**DOI:** 10.3390/cancers15082240

**Published:** 2023-04-11

**Authors:** Lijie Xing, Yuntong Liu, Jiye Liu

**Affiliations:** 1 Department of Hematology, Shandong Cancer Hospital and Institute, Shandong First Medical University and Shandong Academy of Medical Sciences, Jinan 250117, China; 2 Jerome Lipper Multiple Myeloma Center, LeBow Institute for Myeloma Therapeutics, Dana-Farber Cancer Institute, Harvard Medical School, Boston, MA 02215, USA

**Keywords:** B cell maturation antigen, multiple myeloma, antibody-drug conjugates, Anti-BCMA ADC, drug resistance

## Abstract

**Simple Summary:**

Despite the variety of drugs used to treat multiple myeloma and the ever-lengthening survival times, a recurring problem is that many current drugs affect healthy cells, causing side effects, additional illnesses, and drug intolerances. In theory, antibody-drug conjugates targeting deliberately chosen antigens can deliver drugs directly to cancer cells with minimal damage to healthy cells. As such, the conjugates targeting the B cell maturation antigen, which is restrictively expressed on malignant plasma cells, are an active area of research. Several of them are currently in clinical trials to test their safety and efficacy, both alone and in combination with other therapies. The findings will provide better treatment options for multiple myeloma patients.

**Abstract:**

Multiple myeloma (MM) is an incurable cancer of the plasma cells. In the last twenty years, treatment strategies have evolved toward targeting MM cells—from the shotgun chemotherapy approach to the slightly more targeted approach of disrupting important MM molecular pathways to the immunotherapy approach that specifically targets MM cells based on protein expression. Antibody-drug conjugates (ADCs) are introduced as immunotherapeutic drugs which utilize an antibody to deliver cytotoxic agents to cancer cells distinctively. Recent investigations of ADCs for MM treatment focus on targeting B cell maturation antigen (BCMA), which regulates B cell proliferation, survival, maturation, and differentiation into plasma cells (PCs). Given its selective expression in malignant PCs, BCMA is one of the most promising targets in MM immunotherapy. Compared to other BCMA-targeting immunotherapies, ADCs have several benefits, such as lower price, shorter production period, fewer infusions, less dependence on the patient’s immune system, and they are less likely to over-activate the immune system. In clinical trials, anti-BCMA ADCs have shown safety and remarkable response rates in patients with relapsed and refractory MM. Here, we review the properties and clinical applications of anti-BCMA ADC therapies and discuss the potential mechanisms of resistance and ways to overcome them.

## 1. Introduction

Multiple myeloma (MM) is a hematological malignancy that ranks as the second most frequent, representing 1% of all cancers [1,2]. MM is characterized by neoplastic plasma cell proliferation, which leads to the accumulation of monoclonal plasma cells in the bone marrow and the excess production of M protein, ultimately resulting in end-organ damage [3]. Before the 21st century, the mainstay of MM treatment was chemotherapy [4]. However, in recent decades, several therapeutic agents, including proteasome inhibitors (PIs, e.g., bortezomib), immunomodulatory drugs (IMiDs, e.g., thalidomide, lenalidomide, and pomalidomide), and histone deacetylase inhibitors (HDACis, e.g., panobinostat), have been developed to treat MM. These drugs prolonged the overall survival (OS) of MM patients [5,6,7,8,9], but MM remains an incurable disease because of the eventual emergence of drug resistance, causing many MM patients to relapse [10]. Patients with newly diagnosed MM have a 5-year OS rate of 76.1% [11], but those who are refractory to PIs and IMiDs have a dismal median OS of 13 months [12].

PIs and IMiDs perturb pathways that are important for MM cell survival, but they also affect a broad range of other cells. Therefore, immunotherapy strategies have been developed to target highly expressed MM antigens, such as signaling lymphocytic activation molecule F7 (SLAMF7) and CD38 [13,14,15,16,17]. Despite promising early clinical results using the corresponding monoclonal antibodies (mAbs, e.g., elotuzumab and daratumumab) [16,18,19,20,21,22,23,24], these therapies lead to non-specific toxicities since these antigens are found in other normal tissues [25]. For example, daratumumab rapidly reduces the number of CD38+ natural killer (NK) cells, which impairs MM cell killing [26,27]. A better alternative would be to target an antigen that is specifically expressed in MM cells and is key to MM cell survival.

## 2. B Cell Maturation Antigen (BCMA)

B cell maturation antigen (BCMA) was first identified in 1992 on the short arm of chromosome 16 at 16p13.1 in malignant human T-cell lymphoma [28]. It is a type III transmembrane glycoprotein with 6 conserved cysteines in its extracellular domain. It belongs to the tumor necrosis factor receptor (TNFR) superfamily as *TNRSF17* and is primarily present in a perinuclear structure that overlaps the Golgi apparatus, but functional BCMA is also found on the cell surface [28,29,30,31].

BCMA functions in conjunction with two related TNFR superfamily members, B-cell activation factor receptor (BAFF-R) and transmembrane activator and calcium modulator and cyclophilin ligand interactor (TACI). Their collaboration regulates various aspects of B cell activities, such as proliferation, survival, maturation, and differentiation into plasma cells (PCs) [29,30,32]. Upon binding its cognate ligands, BAFF and APRIL, BCMA can activate the NF-kB, Elk-1, p38, or JNK pathways to transduce signals for corresponding functions [31,33,34,35]. Conversely, a soluble form of BCMA (sBCMA), generated by γ-secretase (GS), neutralizes APRIL as a decoy and hinders the activation of subsequent BCMA pathways [36]. The sBCMA level has been suggested as a biomarker since it is significantly higher in MM patients compared to healthy individuals, and higher levels are associated with poor prognosis [37,38], MM progression, and poor response to BCMA-targeted therapy [38,39].

Since its discovery, various studies have demonstrated that BCMA is a promising immunotherapeutic target for multiple myeloma. BCMA expression is restrictively found on the surface of plasmablasts and differentiated PCs, with no expression on CD34+ hematopoietic stem cells, naive B cells, memory B cells, and other normal tissue cells [40]. Furthermore, it has a high expression on the surface of the MM cell and is necessary for the survival of long-lived bone marrow plasma cells [41,42]. Both BCMA mRNA and protein have higher expression in malignant PCs than normal PCs, as validated by multiple gene expression profiling [40,43,44] and immunohistochemistry studies [40]. Moreover, Carpenter et al. [40] found BCMA cDNA in several hematologic tissues, including blood leukocytes, bone marrow, spleen, lymph node, and tonsil, but no BCMA cDNA in other normal human tissues except for the testis, trachea, and some gastrointestinal organs, where low levels of BCMA cDNA were detected, likely from plasma cells present in lamina propria and Peyer’s patches. These findings indicate BCMA is a desirable therapeutic target.

## 3. BCMA-Targeted Immunotherapy in MM

Because of the high BCMA expression in malignant PCs, the development of novel therapies targeting BCMA is rapidly progressing. There are three major types of BCMA-targeted therapy currently under pre-clinical and clinical development: bispecific T cell engagers (BiTE), chimeric antigen receptor T cells (CAR-T), and antibody-drug conjugates (ADC). A BiTE employs antibodies with 2 arms to bind both MM cells and CD3 receptors on T cells, which activates T-cells and encourages cancer cell lysing [45]. Its small size makes it highly efficacious in bringing two cells into contact, but it has a short half-life that requires continuous infusions [46]. The effectiveness of the treatment is dependent on the patient’s T cell function, which works best when the patient has limited prior treatments and a minimal disease burden. CAR-T is the adoptive transfer of T cells genetically modified to recognize tumor-associated antigens [47]. The first anti-BCMA CAR-T was synthesized in 2013 [40], and in the phase 1 clinical trial, it caused remission of MM [39]. Currently, two anti-BCMA CAR-T therapies have been approved by the FDA for patients with R/R MM. However, preparing CAR-T cells takes several weeks, during which patients may need bridging therapies to control their malignancy. The high cost also limits the application of CAR-T therapy. In addition, both BiTE and CAR-T cells are associated with T cell activation, which may induce overactive immune responses, including cytokine release syndrome (CRS) and hemophagocytic lymphohistiocytosis/macrophage activation syndrome (HLH/MAS). Neurotoxicity is another common serious adverse event induced by this therapy [48].

Even though the development of BCMA-targeted therapies is unprecedented, a common issue with these therapies is the occurrence of hypogammaglobulinemia which may result in a high risk of infection [49]. This side effect arises from the “on-target, off-tumor” effect of targeting BCMA, as the normal plasma cells expressing BCMA are also eliminated by the treatments. Hypogammaglobulinemia and associated infections have been reported and investigated in multiple anti-BCMA CAR-T and BiTE studies [49,50,51,52,53], indicating a low occurrence rate but a profound and lasting impact. To manage this issue, immunoglobulin replacement therapy is utilized.

## 4. Anti-BCMA ADC

An antibody-drug conjugate (ADC) solves many of the above problems. An ADC comprises three components: a monoclonal antibody against a target, a cytotoxic agent (payload), and a stable linker connecting the two [54,55,56]. Since only a small amount of injected antibodies localize to tumor cells, most payloads are highly potent, with cytotoxicity in the picomolar range, often targeting tubulin or causing DNA damage [56,57,58]. The linker covalently binds the payload to the antibody and is critical to ADC efficacy, pharmacokinetics, pharmacodynamics, and therapeutic index. A stable linker ensures the release of the cytotoxic drug to target tissue and minimizes toxic effects. On the other hand, an overly strong linker impedes the delivery of the drug. Both cleavable and non-cleavable linkers, which rely on the physiological environment and degradation in endosomes and lysosomes, respectively, have been developed [59,60].

**Figure 1:**
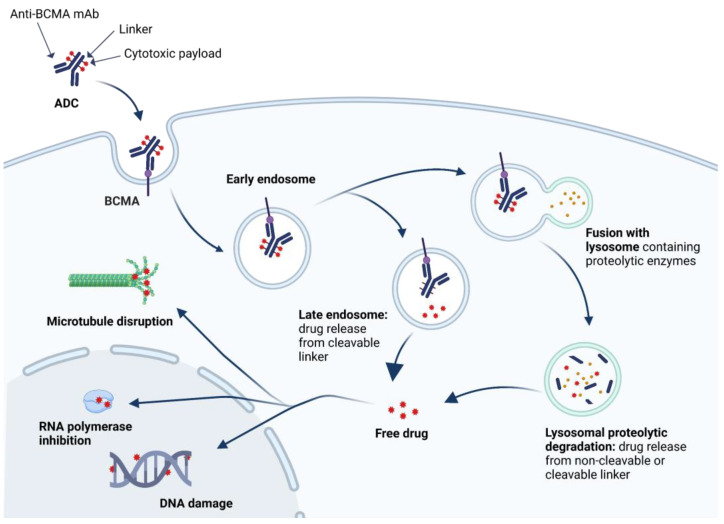
General mechanism action of an anti-BCMA ADC in myeloma cell. Created with BioRender.com.

In MM treatment, BCMA is considered one of the most promising targets of ADCs. Following binding to BCMA, the ADC is internalized by endocytosis. The drug is released by cleavage or degradation in endosomes or lysosomes and then causes DNA damage, inhibits transcription, or disrupts microtubules, which leads to apoptosis (Figure 1). The development of anti-BCMA ADCs is an active area of research, and several anti-BCMA ADCs are in various stages of clinical trials (Table 1).

### 4.1. Belantamab Mafodotin

Belamaf (J6M0-mc–MMAF, belantamab mafodotin, GSK2857916) is the most well-studied ADC in myeloma. Its antibody component is an afucosylated IgG1 directed to BCMA (Kd of ~0.5 nM) [43]. Once it binds BCMA on the MM cell membrane, the entire ADC is internalized and digested in the lysosome, which breaks the non-cleavable maleimidocaproyl (MC) linker and releases the drug monomethyl auristatin F (MMAF). MMAF blocks tubulin polymerization and induces G2-M growth arrest, thus causing caspase 3/7-dependent apoptosis [43]. MMAF is a synthetic analog of dolastatin, a common drug component in ADCs. Its non-cell-permeable nature reduces the toxicity to healthy cells.

The benefits of Belamaf are not limited to its ability to induce apoptosis directly. The NF-kB signaling pathway essential for MM cell growth and survival is blocked by Belamaf as its specifically engineered anti-BCMA antibody competes with APRIL and BAFF for binding to BCMA [43]. It is worth noting that blocking BAFF and APRIL can impair the function of immune cells, such as T cells and NK cells, and potentially lead to an increased susceptibility to infections and other diseases [77]. It was also noted that the afucosylation of its Fc domain substantially enhances the binding affinity to the FcγR (FcγRIIIa) present in effector cells, such as NK cells, monocytes, and macrophages. Consequently, the killing of MM cells is further improved by elevated antibody-dependent cell-mediated cytotoxicity (ADCC) and antibody-dependent cellular phagocytosis (ADCP).

The multicenter DREAMM-1 phase I (NCT02064387) was the first in-human trial of Belamaf. This dose-escalation trial was conducted in 38 MM patients who were relapsed/refractory to daratumumab, PI, and/or IMiDs [61]. The drug was given by intravenous infusions once every 3 weeks, starting at 0.03 mg/kg and increasing to 3.4 mg/kg. No dose-limiting toxic events were observed; thus, no maximum tolerated dose was established. The overall clinical benefit rate was 25%. The most common side effects were nausea (97%), fatigue (47%), thrombocytopenia (45%), anemia (42%), vision blur (29%), chills (24), dry eye (24%), aspartate aminotransferase increase (21%), and pyrexia (21%). Five patients (14%) were considered to have experienced severe adverse events (SAEs) due to the treatment. Two patients had infusion-related reactions. The other three patients had intracranial hemorrhage, lung infection and pyrexia, or pericardial effusion. According to these results, the dose-expansion trial of DREAMM-1 treated 35 advanced R/R MM patients with 3.4 mg/kg Belamaf once every 3 weeks. The median time to the first response was 1.4 months. The results were promising, with an overall response rate of 60% (21 patients), 3% stringent complete response (sCR), 6% complete response (CR), 43% very good partial response (VGPR), and 9% partial response (PR). Most adverse events were grade 1–2, with 31 (89%) of 35 patients having corneal events in the ophthalmological examination, which were characterized by superficial punctate keratitis (77%) associated with epithelial (microcystic) edema (63%), stromal edema (14%), or opacities (23%). Grade 3/4 adverse events were mainly thrombocytopenia (35%) and anemia (14%). Treatment-related serious adverse events occurred in more than 2 patients. The update in 2019 reported a median progression-free survival (mPFS) of 12 months and a median duration of response of 14.3 months [62]. From both phases 1 and 2, a total of 12 treatment-related serious adverse effects and no treatment-related deaths were reported. In this trial, the corneal events appeared frequently, and the frequency increased in the higher Belamaf dose setting. This was attributed to the off-target damage by MMAF to corneal epithelial cells, which was noted in the animal study [78]. The visual symptoms were mild and manageable but caused many dose adjustments and even treatment discontinuations. Nonetheless, these safety and efficacy results supported the approval of Belamaf as a Breakthrough Therapy by the FDA and as a priority medicine (PRIME) by the European Medicines Agency (EMA) in 2017.

Because of the association between Belamaf dosage and ocular events, DREAMM-2 (NCT03525678) was carried out as an open-label, randomized, phase II, two-arm study comparing the safety and efficacy of two different dosages of Belamaf [63]. The trial recruited heavily pre-treated R/R MM patients who were refractory to IMiDs and PIs. Additional recruitment requirements emphasized being refractory and/or intolerant to anti-CD38 mAb. 196 MM patients were included and randomized to receive 2.5 mg/kg (n = 97) or 3.4 mg/kg (n = 99) Belamaf via intravenous infusion once every 3 weeks. The most recent report found a 31% overall response rate (ORR) in the 2.5 mg/kg cohort and 35% ORR in the 3.4 mg/kg cohort [64]. The duration of response (DoR) was either not reached or was 6.2 months in the 2.5 mg/kg and 3.4 mg/kg groups, respectively. The estimation of 1-year OS was 53%. The most common grade 3/4 adverse events in the safety population were keratopathy (29% vs. 24%), thrombocytopenia (21% vs. 32%), anemia (20% vs. 27%), and neutropenia (11% vs. 16%). Each cohort reported one death that might be associated with the treatment. The one in the 2.5 mg/kg cohort was caused by sepsis, and the other in the 3.4 mg/kg cohort was due to haemophagocytic lymphohistiocytosis. The follow-up was extended after 13 months to examine safety and efficacy in the longer term. Until this extended follow-up, 10% of patients were still receiving Belamaf at 2.5 mg/kg. The estimated mDoR, OS, and PFS were 11.0 months, 13.7 months, and 2.8 months. The clinical activity was sustained without new adverse events [65]. This trial also examined the mitigation strategies for Belamaf-related ocular events in an ocular substudy, which includes the use of corticosteroid eye drops and preservative-free artificial tears and the application of a cooling eye mask before infusion in addition to treatment adjustments [78]. It was found that the best strategies were to use artificial tears. Because of the clinically meaningful anti-myeloma activity and manageable safety profile of Belamaf from the first report, it was approved by the FDA in 2020 as a monotherapy treatment for R/R MM patients who have received 4 prior therapies including anti-CD38 mAb, PI, and IMiDs.

The phase III, open-label, randomized, two-arm study DREAMM-3 (NCT04162210) was conducted to compare the effect of single-agent Belamaf vs. pomalidomide (Pom) plus low-dose dexamethasone (Dex). Recruited patients had R/R MM and were treated with more than 2 prior lines of therapy [66]. The study randomized 325 patients in a 2:1 ratio to receive either 2.5 mg/kg of Belamaf once every three weeks or Pom daily with Dex weekly. The primary analysis was released in November 2022, and its primary endpoint of PFS was not met [67]. At the time of the analysis, the median OS was 21.2 months and 21.1 months, and the mPFS was 11.2 months and 7 months for the Belamaf group and Pom + Dex group, respectively. The Belamaf cohort had an ORR of 41%, while the Pom + Dex cohort had an ORR of 36%. Belamaf showed a better response rate than Pom + Dex (25% VGPR vs. 8% VGPR). DoR rates at 12 months were 76.8% and 48.4%. It was mentioned that the overall rates of grade 3 adverse events of keratopathy were comparable with previously published data, but no statistics of AEs were reported in this update. No new treatment-related safety issue was identified. Based on the outcome of the DREAMM-3, GSK initiated the process for withdrawal of the US marketing authorization for Belamaf on November 2022, but would continue trial programs for the drug.

The potential of combining Belamaf with standard and novel treatments to treat R/R MM is being intensively investigated in various phase I, II, or III clinical trials. The phase I/II study, ALGONQUIN (NCT03715478), explored the safety and efficacy of Belamaf in combination with Pom and Dex (Pd) in R/R MM patients pre-treated with lenalidomide and a PI [68]. The initial dose-escalation study identified the maximum tolerated dose as 2.5 mg/kg Belamaf combined with a standard dose of Pd. Although all dosing cohorts showed deep and durable responses, the 2.5 mg/kg Belamaf combination treatment appeared to have the best efficacy with 89.8% ORR, 77% at least VGPR, and 90% at 6 months PFS. In DREAMM-4 (NCT03848845), Belamaf was combined with pembrolizumab (anti-PD-L1) [69], and the primary analysis demonstrated a favorable ORR compared with only Belamaf in heavily pre-treated R/R MM patients [70]. DREAMM-5 (NCT04126200) contains multiple substudies to evaluate different Belamaf-containing combinations, including the T-cell activating checkpoint mAbs GSK3359609 and the GS inhibitor nirogacestat which increases BCMA level on the myeloma cell surface [71]. Preliminary dose exploration and cohort expansion results from the low-dose Belamaf + nirogacestat substudy showed encouraging clinical activity, with 38% ORR (9/24) and 17% (4/24) VGPR in both phases combined [72]. The safety profile of this combination treatment was reported to be manageable in heavily pre-treated R/R MM. DREAMM-6 (NCT03544281) assessed the combination of Belamaf with lenalidomide (Len) plus Dex (arm A) or bortezomib plus Dex (arm B) [73]. At interim follow-up, the ORR was 78%, with 50% VGPR and a clinical benefit rate of 83%.

Currently, the efficacy and safety of the Belamaf combination regimen are being compared to the approved standard-of-care therapy for R/R MM patients. In particular, Belamaf combined with bortezomib plus Dex will be compared to daratumumab alone (DREAMM-7, NCT04246047), and Belamaf combined with Pd will be compared to bortezomib with Pd (DREAMM-8, NCT04484623). Additionally, in transplant-ineligible newly diagnosed MM patients, DREAMM-9 (NCT04091126) will compare the efficacy and safety of Belamaf in combination with bortezomib, lenalidomide, and dexamethasone (VRd) versus VRd alone. The results from these clinical trials will determine the benefit of adding Belamaf to the standard of care.

### 4.2. MEDI2228

MEDI2228 includes a fully humanized BCMA antibody conjugated to pyrrolobenzodiazepine (PBD) via a protease-cleavable linker. In pre-clinical models, this ADC targets bulk MM cells as well as patient MM progenitor cells that are CD19 + CD138- and kills cells by inducing multiple DNA damage response genes via phosphorylating ATM/ATR kinases, checkpoint kinases 1/2 (CHK1/2), and H2AX regardless of p53 status [79]. Unlike Belamaf, MEDI2228 preferentially binds to membrane-bound BCMA over sBCMA [80], which makes it more efficient than Belamaf and means its cytotoxicity is minimally affected by sBCMA levels.

The first-in-human phase I trial of MEDI2228 (NCT03489525) was conducted in 82 R/R MM patients who had progressed after treatment with three standard-of-care MM drugs, PIs, IMiDs, and mAbs [74]. MEDI2228 was infused intravenously at incrementally increasing doses (0.0125, 0.25, 0.05, 0.1, and 0.2 mg/kg) once every 3 weeks. The maximum tolerated dose was 0.14 mg/kg due to dose-limiting toxicities. Several adverse events were observed in the 0.14 mg/kg cohort, with photophobia being the most common (54%), followed by thrombocytopenia (32%), rash (30%), elevated gamma-glutamyl-transferase (24%), dry eye (20%), and pleural effusion (20%). Notably, there were no cases of keratopathy or visual acuity loss. All dosage levels were demonstrated to be effective in this study. Nonetheless, the most favorable response was from the 0.14 mg/kg cohort, which had the highest ORR (61%), and no median DOR was reached. It is worth mentioning that in this cohort, 90% of the patients received prior daratumumab treatment. Therefore, MEDI2228 demonstrated impressive single-agent clinical activity in heavily pre-treated MM patients that had received previous immunotherapy.

In vitro and in vivo studies suggest that MEDI2228 has a synergistic effect with bortezomib and DNA damage response checkpoint inhibitors [81]. Furthermore, as MEDI2228 upregulates expression of CD38 and NKG2D ligands on the MM cell surface, it increases NK cell immune activity and restores daratumumab-induced ADCC, supporting the combination of CD38- and BCMA-targeted immunotherapies [82].

### 4.3. AMG 224

AMG 224 is composed of an anti-BCMA antibody, the non-cleavable linker 4-(N-maleimidomethyl) cyclohexane-1-carboxylate (MCC), and the cytotoxic agent DM1. DM1, a derivative of the ansamycin antibiotic maytansine, is a tubulin development inhibitor that prevents tumor growth [83]. The clinical activity of AMG 224 was tested in a phase I clinical trial (NCT02561962) with R/R MM patients who had heavy pretreatments, including IMiDs and PI [75]. Twenty-nine patients received AMG 224 in dose escalation (30–250 mg), and 11 patients received AMG 224 in dose expansion (3 mg/kg). The overall ORR was 23%, containing 2 stringent CR, 2 VGPR, and 5 PR. The median DoR in the dose escalation phase was 14.7 months. Similar to Belamaf, the most common grade ≥3 adverse event was thrombocytopenia (40%), but it occurred more often than in the Belamaf phase I trial (9%). Treatment-emergent ocular adverse events were seen in 30% of patients with no dose reduction due to these events, contrasting to 46% of patients who had Belamaf dose reduction because of these. This clinical trial provided proof of the safety and benefits of AMG 224 in R/R MM patients.

### 4.4. HDP-101

HDP-101 is another anti-BCMA ADC, but its cytotoxic agent, amanitin, is a new class of payload that impedes the transcription process by inhibiting RNA polymerase II. This reduces cell proliferation and causes cell apoptosis at very low concentrations. In in vitro MM cell models, the picomolar range of HDP-101 was cytotoxic to BCMA+ cells but not BCMA− cells [84]. In mouse xenograft models, tumor reduction and complete remission were observed depending on the HDP-101 dose. The safety of HDP-101 was further evaluated in nonhuman primates, where only a transient, mild to moderate increase in liver enzymes and lactate dehydrogenase was observed, indicating good tolerability and therapeutic index. Another recent study showed that it suppressed tumor burden in cell lines with a 17p deletion, which remains an adverse prognostic factor of MM [85].

A phase I/II clinical trial of HDP-101 (NCT04879043) is currently enrolling patients with progressed MM [76]. Preliminary safety data from 4 patients were presented at the American Society of Hematology (ASH) 2022 meeting, and they found no keratopathy or decrease in visual acuity. No free payload was found in pharmacokinetic samples, and the first 2 dose cohorts showed good tolerability to HDP-101. Higher dose cohorts will be evaluated after the enrollment of new patients.

### 4.5. CC-99712

CC-99712 is an anti-BCMA ADC granted orphan drug designation by the FDA in 2021. Its payload is a non-cleavable maytansinoid. To date, no pre-clinical study has been published concerning this ADC. A phase I clinical trial (NCT04036461) is currently ongoing in R/R MM patients, but no results have been posted.

## 5. Mechanisms of Resistance to anti-BCMA ADC and Strategies to Overcome

**Figure 2:**
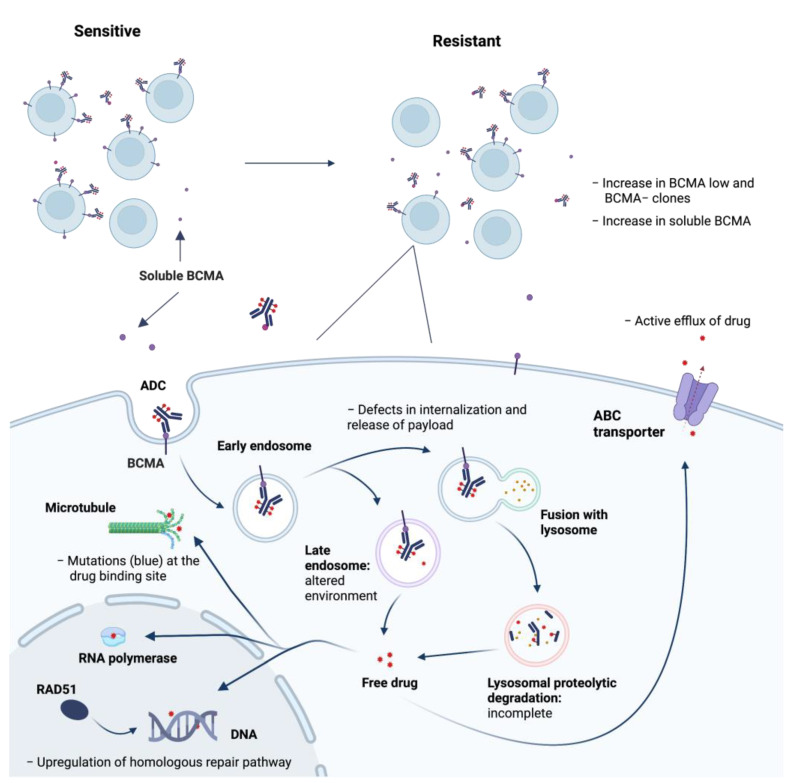
General resistance mechanism of anti-BCMA ADC in treating multiple myeloma. Created with BioRender.com.

The development of treatment resistance is a continual problem faced by myeloma patients and indicates poor prognosis [86]. Due to the recent inclusion of anti-BCMA ADCs in the treatment of MM, the mechanisms underlying intrinsic and acquired resistance to these drugs are still poorly understood [87]. However, several mechanisms have been proposed based on the pathways that ADCs rely upon (Figure 2) [88].

First, an ADC must attach to a myeloma cell by binding BCMA. Therefore, the loss or downregulation of BCMA on myeloma cells is predicted to cause resistance. As MM is a heterogeneous disease with a diversity of subclones, it is possible that BCMA^low^ or BCMA– cells would gain growth advantages during anti-BCMA ADC therapy. This is evident during BCMA-specific CAR-T therapy, in which the loss of cell-surface BCMA from myeloma cells was observed [89]. In addition, the high sBCMA levels in many MM patients would decrease the efficacy of anti-BCMA ADCs since the sBCMA sequesters the anti-BCMA antibody and prevents targeting to MM cells.

The loss of BCMA can be circumvented by incorporating new formats of antibodies into ADCs. A biparatopic or bispecific antibody could improve targeting by enhancing recognition of the same or two separate antigens. In relation to sBCMA, MEDI2228 shows strong binding to membrane BCMA, with levels up to 720 ng/mL of sBCMA having little impact on the IC_50_ of MEDI2228 [80]. Moreover, small-molecule GS inhibitors (GSis) can increase surface BCMA levels by reducing the cleavage of BCMA from the membrane, subsequently improving MM cell recognition by anti-BCMA ADCs. In fact, GSis improve the efficacy of anti-BCMA CAR-T therapy in vivo [90]. Accordingly, a clinical trial combining GSis with concurrent anti-BCMA CAR-T therapy has been initiated (NCT03502577), which could inform on their ability to enhance anti-BCMA ADC therapy.

After binding BCMA, the ADC is transported into the cell by clathrin-mediated (CME), caveolin-mediated, or clathrin-caveolin-independent endocytosis. For ADCs with cleavable linkers, cleavage might occur in the endosome. If not, the endosome fuses with a lysosome [91], which catalyzes complete ADC degradation and payload release [88,92]. If any of these processes are impaired, the payload will not be released and cannot perform its cytotoxic function. In fact, in cells resistant to the ADC T-DM1, an altered pH within the lysosomes prevents the degradation of T-DM1 [93]. Switching out the linker to one that is protease-cleavable solves this issue in at least 2 cell models of T-DM1 resistance [94].

Even if the payload is released, it must stay within the cell to perform its function. As we’ve learned from other MM drugs, the ATP binding cassette (ABC) transporters reduce the effectiveness of small molecule chemotherapeutic agents by actively eliminating the agent from the cytoplasm [95]. It is likely that the conjugated cytotoxins are substrates of ABC transporters, and this could result in resistance. This can be solved by changing the payload to one with a low affinity to efflux pumps. For example, vadastuximab talirine, an anti-CD33 ADC, uses pyrrolobenzodiazepines (PBD) as the cytotoxic agent instead of its original payload, gemtuzumab ozogamicin (GO), because PBD is a poor substrate for drug efflux pumps. In an in vitro study, PBD demonstrated anti-leukemia activity in multidrug-resistant acute myeloid leukemia (AML) models, including those resistant to GO [96]. Another strategy is to reduce the hydrophobicity of the cytotoxic compound because hydrophobic compounds are good substrates for multidrug resistance protein (MDR) transporter. In a study with antibody-maytansinoid conjugates, a hydrophilic metabolite of maytansinoid was produced using a hydrophilic linker and was more potent in killing MDR-expressing cells than other metabolites from nonpolar linkers [97].

If an ADC succeeds in binding an MM cell, releasing its payload, and avoiding active efflux, the cell might still survive the drug by activating survival pathways. In MM, drug resistance is often linked to activated DNA repair pathways [98,99]. One of the DNA repair pathways, the homologous recombination pathway (HR), recruits a protein called RAD51 [100]. This protein plays a key role in the repair of DNA lesions, including interstrand cross-links, stalled/damaged replication forks, and double-strand breaks. Cells with increased RAD51 levels evade radiation- or chemotherapy-induced DNA damage and acquire resistance leading to poor patient survival [101]. In the case of ADC treatment, the upregulation of RAD51 and increase in HR were observed in the clinical trial with MEDI2228 in MM. The addition of DNA damage repair checkpoint inhibitors, such as AZD0156 (ATMi), AZD6738 (ATRi), and AZD1775 (WEE1i), synergized with MEDI2228 to enhance cytotoxicity [102].

Finally, ADC resistance could occur from mutations in the targets of the cytotoxic drugs. However, no mutations have been found in tubulin, topoisomerase I, or RNA polymerase II in any ADC-resistant models [87].

## 6. Conclusions

BCMA-based immunotherapy has shown potential as a therapy for MM. In multiple clinical trials, anti-BCMA ADCs have proven safe and effective, even in heavily pre-treated R/R MM patients. Nonetheless, there is still much to be learned about the optimal use of these agents, including patient selection, dosing strategies, and combination treatments. Moving forward, we anticipate continued development and refinement of anti-BCMA ADCs using next-generation antibodies and drugs. Although we are just at the very beginning of recognizing anti-BCMA ADC resistance in myeloma, the theoretical resistance mechanisms based on ADC structure and function have provided directions for therapeutic intervention.

## Figures and Tables

**Table 1:** Summary of current clinical trials of anti-BCMA ADC.

Drug	Clinical Trial	Prior Lines of Therapy (Median)	N	Results	Most Common AEs	Ref.
Belamaf	DREAMM-1 (NCT02064387), phase I	≥2	73	Dose expansion part (n = 25): ORR 60%, CR 3%, 43% VGPR, 9% PR, mPFS 12 months, mDoR 14.3 months	Nausea, fatigue, thrombocytopenia, anemia, vision blur, chills, dry eye, aspartate aminotransferase increase, pyrexia	[61,62]
Belamaf (2.5 vs. 3.4 mg/kg)	DREAMM-2 (NCT03525678), phase II	≥3	197	ORR 31% vs. 35%, ≥VGPR 19% vs. 24%, mDoR NR vs. 6.2 months, mPFS NR vs. 8.4 months	Keratopathy, thrombocytopenia, anemia, neutropenia	[63,64,65]
Belamaf vs. Pd	DREAMM-3 (NCT04162210), phase III	≥2	325	ORR 41% vs. 36%, ≥VGPR 25% vs. 8%, mPFS 11.2 months vs. 7 months, does not meet the primary endpoint of PFS, mOS 21.2 months vs. 21.1 months		[66,67]
Belamaf + Pd	ALGONQUIN (NCT03715478), phase I/II	1–5 (3)	60	ORR 88.9%, ≥VGPR 74.1%, mPFS 24.2 months	Keratopathy, blurred vision, fatigue, neutropenia, thrombocytopenia, fever, diarrhea, constipation, dry eye	[68]
Belamaf + pembrolizumab	DREAMM-4 (NCT03848845), phasen I/II	3–13 (5)	34	ORR 47%, CR 12%, VGPR 18%, PR 18%, mDoR 8.0 months, mPFS 3.4 months	Keratopathy, blurred vision, thrombocytopenia	[69,70]
Belamaf-containing combinations (e.g., Belamaf + nirogacestat)	DREAMM-5 (NCT04126200), phase I/II	3–10 (4.5)	10	ORR 60%, VGPR 20%, PR 40%	Ocular events	[71,72]
Belamaf + Len + Dex vs. Belamaf + Bortezomib + Dex	DREAMM-6 (NCT03544281), phase I/II	1–11 (3)	45	ORR 78%, ≥VGPR 50%	Keratopathy, blurred vision, dry eye, thrombocytopenia	[73]
Belamaf + Bortezomib + Dex vs. Daratumumab + Bortezomib + Dex	DREAMM-7 (NCT04246047), phase III		575 (estimated)			
Belamaf + Pd vs. Bortezomib + Pd	DREAMM-8 (NCT04484623), phase III		300 (estimated)			
Belamaf + VRd vs. VRd	DREAMM-9 (NCT04091126), phase I		144 (estimated)			
MEDI2228	NCT03489525, phase I	2–11	82	ORR 61%, VGPR 24.4%, PR 36.6%, DoR not reached	Photophobia, thrombocytopenia, rash, increased gamma-glutamyltransferase, dry eye, pleural effusion	[74]
AMG 224	NCT02561962, phase I	2–11(7)	42	ORR 23%, CR 5%, VGPR 5%, PR 13%, mDoR 14.7 months	Thrombocytopenia, fatigue, nausea, aspartate aminotransderase increase, anemia	[75]
HDP-101	NCT04879043, phase I/II	5–16 (11)	4			[76]
CC-99712	NCT04036461, phase I		160 (estimated)			

Pd: pomalidomide and dexamethasone; Len: lenalidomide; Dex: lexamethasone; VRd: bortezomib, lenalidomide, and dexamethasone; AE: Adverse event; ORR: overall response rate; CR: complete response; VGPR: very good partial response; PR: partial response; mDoR: median duration of response; NR: not reached; mPFS: median progression-free survival; mOS: median overall survival.

## Data Availability

Not applicable.
